# Improved preventive care clinical decision-making efficiency: leveraging a point-of-care clinical decision support system

**DOI:** 10.1186/s12911-021-01675-8

**Published:** 2021-11-11

**Authors:** Scott Laing, Jay Mercer

**Affiliations:** 1 Department of Family Medicine, Primrose Family Medicine Centre, University of Ottawa, Ottawa, Canada; 2 Department of Family Medicine, Bruyère Family Medicine Centre, University of Ottawa, Ottawa, Canada

**Keywords:** Clinical-decision support, Preventive care, Quality improvement

## Abstract

**Background:**

Electronic medical records are widely used in family practices across Canada and can improve health outcomes. However, recent reports indicate that physicians using electronic medical records work longer and have less direct patient contact which may contribute to burnout. Therefore, new and innovative digital tools are essential to reduce physician workloads and improve patient-physician interaction to address physician burnout. The objective of this study was to assess the efficiency and accuracy of clinical decision-making when using a new preventive care point-of-care clinical decision support system (CDSS). An estimate of the potential annual time savings was also determined. This study also assessed physician reported perceived usefulness and ease of use of the CDSS.

**Methods:**

Quantitative and qualitative data were collected during this study. Each participant evaluated two simulated patient charts and identified which preventive care metrics were due. The participants recorded their decisions and the time required to assess each chart. Participants then completed a Technology Acceptance Model survey regarding the perceived usefulness and ease of use of the CDSS, which included qualitative feedback. The amount of time saved was determined and participants’ clinical decision-making accuracy was scored against current Canadian preventive care guidelines. The number of preventive care specific visits completed per year was determined using clinic billing data.

**Results:**

The preventive care CDSS saved an average of 195.7 s of chart review time (249.5 s vs 445.2 s; *P* < 0.001). A total of 1520 preventive visits were performed at Primrose and Bruyère Family Medicine Centres. Extrapolated across the organization, implementation of the new tool could save 82.6 h per year. Decision-making accuracy was not affected by the new tool (78.4% vs 80.9%, *P* > 0.05). Participants rated the perceived ease of use and usefulness to be very high.

**Conclusions:**

New digital tools may reduce providers’ workload without impacting clinical decision-making accuracy. Participants indicated that the preventive care CDSS was useful and easy to use. Further software development and clinical studies are required to further improve and characterize the effect this new CDSS has when implemented in clinical practice.

**Supplementary Information:**

The online version contains supplementary material available at 10.1186/s12911-021-01675-8.

## Background

Preventive care services effectively reduce morbidity, mortality, and overall health care expenditures. In Canada, preventive care services are largely delivered by primary care providers, focusing on screening and counselling on lifestyle risk factors, infectious diseases, metabolic disorders, immunizations, and cancer [1, 2]. Many Canadian primary care physicians perform preventive care services through opportunistic or organized approaches. Opportunistic preventive care occurs as an add-on when patients present for non-preventive care services. Organized preventive care occurs locally within primary care practices via dedicated preventive care visits [3] or regionally/nationally through outreach to individuals [4], like Cancer Care Ontario’s mailed cancer screening letters [5].

Canada Health Infoway—a federally funded non-profit aiming to digitally transform Canada’s health care system—has demonstrated that EMR use improves workflow efficiencies, cost efficiencies, health outcomes, patient safety, and interprofessional communication over paper records [6]. EMRs can make relevant patient data readily available to estimate disease risk [7] and improve preventive care service delivery through alerts that identify patients due for screening [8]. However, many providers override and ignore alerts due to “alert fatigue” where high volumes of irrelevant alerts limit usefulness [9]. As well, only 3–10% of providers use these advanced EMR features [6], which further limits the potential benefits of EMR use on preventive care service delivery.

These purported benefits are especially important for preventive care services since primary care providers do not have sufficient time to complete all recommended services [2]. Yet, recent time motion studies have shown that EMR use consumes significant amounts of clinical time, detracting from patient interaction [10, 11] and higher EMR use is correlated with physician burnout [12]. Burnout may arise from low professional satisfaction caused by poor EMR usability, workflows, and interrupted patient-physician interactions [13]. However, another report suggests negligible impacts on clinician working time [14] and instead frequent task switching may be a major contributor toward perceptions of inefficiency and disruption [15].

With over 85% of Canadian physicians using EMRs in their clinical practice [16] it is therefore prudent to develop better EMR point-of-care tools that facilitate preventive care service deliver while not contributing to alert fatigue or task switching and improving efficiency. Accordingly, this study aims to assess the efficiency, provider decision-making accuracy, ease of use, and usefulness of a proof-of-concept, point-of-care, preventive care CDSS. This preventive care CDSS automatically summarizes recommended preventive care services on a single screen within the EMR, which may be easily opened during dedicated preventive care visits.

## Methods

### Study design

This study followed a pre-post test design in an artificial clinical setting. Participants assessed two artificial patient records and made clinical decisions regarding which preventive care services were due for each patient. One chart review was completed with and one without the preventive care, point of care CDSS. Clinical decision-making accuracy and the time to make these decisions were assessed. Participants also completed a Technology Acceptance Model (TAM) to understand their experience using the automated preventive care CDSS [17]. The EMR used was PS Suites [18].

### Participant recruitment

Participants were recruited from Bruyére Family Health Team in Ottawa, Ontario (n = 18) and completed this study in October 2018. Family Health Teams are interdisciplinary primary care clinics in Ontario, which comprise of physicians, nurses, nurse practitioners, social workers, dieticians and other allied health staff [19]. Our clinic is distributed across two locations in Ottawa. All clinical staff were invited to participate via emailed instructions and data collection forms (Additional files 1–3). All resident physicians were recruited by scheduling time for participation during a lunch time teaching session.

### Point of care CDSS design and artificial chart setup

The preventive care CDSS was designed by SL—a final year family medicine resident physician—within PS Suites EMR [18]. This CDSS allows automated text insertion into a clinical note to facilitate clinical documentation and pulls relevant stored patient data, such as demographics, dates and results of laboratory tests, and patient appointment data [18]. This CDSS was designed following a published preventive care encounter template [1] and the Canadian Task Force on Preventive Health Care guidelines [20] were used to guide development of the CDSS. The CDSS automatically generated a summary note within the EMR and displayed the data for the primary care provider. This summary indicated when each preventive screening test was last completed and the result of each test (when available in EMR’s structured data fields).

Next, two artificial patient charts were created. Both charts represented 65-year-old females and did not contain real patient data. The charts were filled with preventive care data including pap tests, mammograms, type 2 diabetes screening, bone mineral densities, colon cancer screening, and hypertension screening. A 65-year-old female was selected since this age group has the most recommended preventive care services [1, 20]. Irrelevant non-preventive care encounters, laboratory tests, and diagnostic imaging were added to the charts. This irrelevant data was added to create a more complex and robust artificial record, since providers must find relevant clinical data when performing preventive care. The dates of all artificial data (relevant and irrelevant) were varied to avoid participants detecting patterns between the two records, but should have resulted in the same clinical decisions across the two charts.

### Data collection

Participants reviewed the two artificial patient charts and recorded the preventive care metrics that were completed, which tests were due, and the time to complete each chart review. Participants recorded this information on the data collection form (Additional file 2), which also included tests that would not be indicated for 65-years-old female to ensure providers made clinically appropriate decisions. This exercise occurred during in a single session and participants all received the same instruction (no randomization to different groups) due to restricted timelines to complete this resident physician project.

Participants reviewed the first chart and did not have access to the CDSS (usual method). A non-functional version of the CDSS was inserted into the chart that did not extract the patient’s latest preventive care data. Providers had to manually review the chart to complete the exercise and record their findings.

Next, participants reviewed the second chart using the preventive care CDSS (Additional files 4 and 5). Providers could use the summary or search the chart for required information to complete the exercise and record their findings (new method).

Immediately after completing the exercise, participants completed a paper-based TAM survey (Additional file 3) to report the perceived usefulness and ease of use of the CDSS. Then the primary investigator assessed the accuracy of participants’ decision-making by marking participants’ recorded responses from the data collection forms (Additional file 2) according to Canadian preventive care guidelines. The primary investigator performed a thematic analysis of the free-text feedback, assessing the content of positive and negative feedback and categorizing each comment accordingly.

Next, annual time savings during dedicated preventive care visits was determined. The number of dedicated preventive care visits was determined using physician billing codes from the previous year (Dec 9, 2017 to Dec 9, 2018) at Bruyére Family Health Team. Billing codes for dedicated preventive care visits and were summated. This total number of preventive care visits was multiplied by the observed amount of time saved when using the CDSS.

To help explain the time differences, process mapping was used to estimate the minimum number of actions required to complete each simulated chart assessment. Counted actions included keystrokes, clicks, scrolling through search results, reading results, interpreting results, and decision-making. This was mapped by SL only due to project timelines and the time to complete each step was not recorded. SL is a resident physician and regularly performs dedicated preventive care visits. The actions participants performed during their reviews could not be captured based on time and personnel limitations.

### Statistical analysis

The results of 17 participants were included in the statistical analysis. One participant was excluded due to incomplete data. Both the accuracy of clinical decision-making and the time required to complete the chart review were analyzed with a paired, two-tailed *T* test. Potential time savings for preventive care visits were calculated by multiplying the number of preventive care visits completed in the last year by the mean time savings observed. Minimum number of actions required were counted from the process mapping. Perceived usefulness and ease of use scores were calculated by determining both the median and mean. Ninety-five percent confidence intervals were calculated for the mean clinical decision-making accuracy, time savings, perceived usefulness, and ease of use scores. All statistical analysis was completed with Microsoft Excel.

### Ethics

This study was submitted to the University of Ottawa Department of Family Medicine for review and approval prior to commencing. The Department of Family Medicine determined this project was deemed to be quality improvement and therefore a Research Ethics Board application was not required. The Department of Family Medicine’s scholarly project guidelines were followed throughout this project. Participants were informed of the study purpose before verbally consenting to participate.

## Results

### Participant sample

Of the 86 healthcare providers at the Bruyére Family Health Team, 18 participated in the study (20.6%). The 17 participants that were included in the analysis consisted of: 1 medical student, 11 residents, 1 nurse practitioner, 4 staff physicians. No registered nurses participated (Table 1).

**Table 1:** Demographics of eligible participants, number of participants, percent of participants within each group, and percent of participants for each group from total of each at Primrose and Bruyére Family Medicine Centres

Provider type	No. practice wide	No. participants	Percent of participants	Percent of practice that participated
Medical student	1	1	5.6	1.1
Resident physician				
PGY1	47	4	22.2	4.6
PGY2		8	44.4	9.2
Nurse practitioner	4	1	5.6	1.1
Registered nurse	13	0	0	0
Physician	22	4	22.2	4.6
Total	87	18	100	20.6

*PGY* post graduate year indicating level of training

### Mean time to complete chart review

**Fig. 1:**
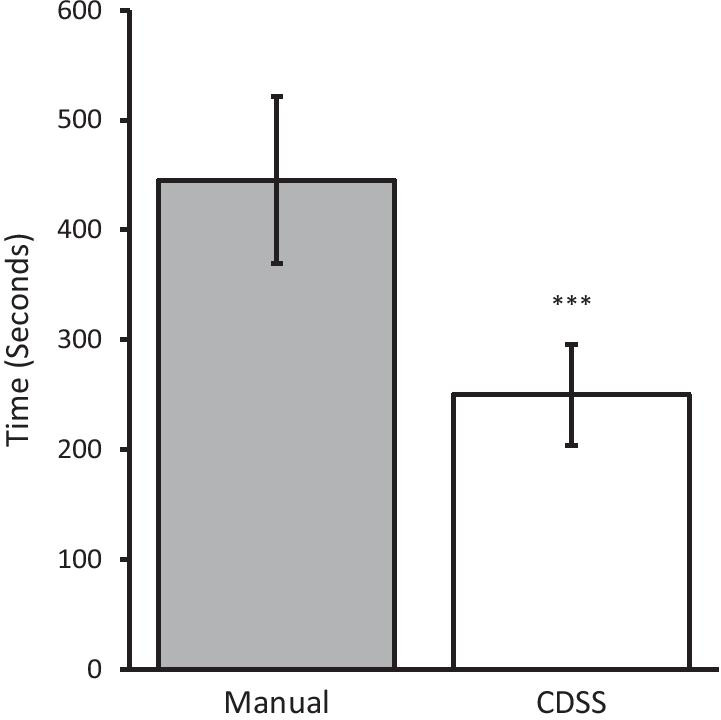
Mean time (± 95% CI) to complete each chart review. Manual and CDSS indicate when participants manually searched the charts and used the preventive care CDSS (clinical decision support system), respectively. ***P < 0.001 (n = 17)

The mean time to complete the chart review with the CDSS was significantly faster than manual chart review (249.5 ± 45.6 s; 95% CI vs 445.1 ± 75.0 s; 95% CI. *P* < 0.001; Fig. 1). The mean time saved during chart review was 195.6 ± 60.4 s; 95% CI.

### Mean clinical decision-making accuracy

**Fig. 2:**
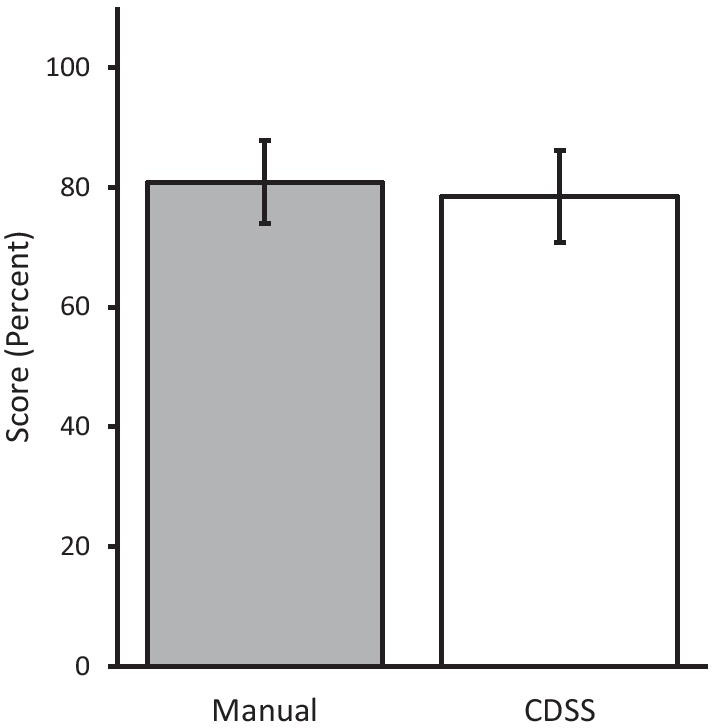
Mean score (percent correct ± 95% CI) of clinical decisions of all participants. Manual and CDSS indicate when participants manually searched the charts and used the preventive care CDSS (clinical decision support system), respectively. *P* = 0.41 (n = 17)

Participants’ decision-making accuracy did not significantly differ between the CDSS and manual chart review (78.4 ± 7.6%; 95% CI vs 80.9 ± 7.0%; 95% CI. *P* = 0.41; Fig. 2).

### Potential time savings for dedicated preventive care visits at Bruyére Family Health Team

In the 365 days preceding this study, a total of 1520 dedicated preventive care visits were completed by Bruyére Family Health Team. The estimated annual clinical time savings from using the preventive care CDSS for these visits could be 82.6 ± 25.5 h per year (95% CI).

### Minimum number of actions required to complete chart review

The process mapping demonstrated manual chart review required 128 actions. In comparison, the CDSS required 58 actions to complete the chart review. Therefore, participants may have performed 70 fewer actions using the preventive care CDSS, explaining some of the time savings.

### Perceived usefulness and ease of use

Participants indicated that the new preventive care tool was both useful (Table 2) and easy to use (Table 3) as represented by median scores of 7/7 across all TAM metrics. The mean scores of perceived usefulness and ease of use were also very high (Tables 2 and 3). The usefulness statement that received the highest score was that “Using this interface would make it easier to do my job” (mean 6.69 ± 0.38; 95% CI out of 7). The ease of use statement that received the highest score was that “Learning to operate this interface would be easy for me” (mean 6.50 ± 0.43; 95% CI out of 7).

**Table 2:** Median and mean perceived usefulness scores from TAM [31]

Statement	Median	Mean	95% CI
Using this interface in my job would enable me to accomplish tasks more quickly	7	6.50	6.04–6.96
Using this interface would improve my job performance	7	6.25	5.74–6.76
Using this interface in my job would increase my productivity	7	6.25	5.74–6.76
Using this interface would enhance my effectiveness on the job	7	6.38	5.95–6.80
Using this interface would make it easier to do my job	7	6.69	6.31–7.06
I would find the new system useful in my job	7	6.63	6.20–7.04

Scores are on scale of 1–7 (n = 17)

*TAM* technology acceptance model

**Table 3:** Median and mean perceived ease of use score from TAM [31]

Statement	Median	Mean	95% CI
Learning to operate this interface would be easy for me	7	6.50	6.07–6.93
I would find it easy to get this interface to do what I want it to do	7	6.25	5.74–6.76
My interaction with this interface would be clear and understandable	7	6.13	5.55–6.70
I would find this interface to be flexible to interact with	7	5.93	5.27–6.59
It would be easy for me to become skillful at using this interface	7	6.19	5.63–6.74
I would find this interface easy to use	7	6.31	5.80–6.82

Scores are on scale of 1–7 (n = 17)

*TAM* technology acceptance model

Qualitative feedback indicated that the preventive care CDSS was perceived to be efficient (70.6%), comprehensive (64.7%), organized (58.8%), and assisted clinical decision making (41.1%) (Table 4). Participants expressed also expressed that the use of “months since last done” was difficult to interpret (41.1%), participants required prior knowledge of preventive care guidelines (23.5%), uncertainty regarding the accuracy of information presented in the CDSS as some participants wanted to see the original reports (17.6%), loss information depth as some measures like pneumococcal vaccination did not show the different types of vaccines (11.8%), and that it was cumbersome to use (11.7%) (Table 5).

**Table 4:** Themes of positive comments regarding new interface

Theme	Percent	Sample comments
Efficient	70.6	“Eliminates need for searching for data”
Comprehensive	64.7	“All results in one place”
Organized	58.8	“Clearly laid out”
Assists Decision Making	41.2	“Time since last done feature is helpful”

Indicates the percentage of participant’s comments that fit into each of the above themes. Sample comments from the feedback are included

**Table 5:** Themes of negative comments regarding new interface

Theme	Percent	Sample Comments
Months since last done difficult to interpret	41.2	“Would be nice to have years next to months if result > 12 months old”
Presumes prior knowledge	23.5	“Presuming[sic] that providers know screening risk, evidence, recommendations”
Concerns re: accuracy	17.6	“May find it hard to trust reported last date without looking at actual report”
Loses depth of information	11.8	“…change to pneu-17 + pneu-23 and 2 types of zoster [vaccinations]”
Cumbersome layout	11.8	“Long list, takes up whole screen”

Indicates the percentage of participant’s comments that fit into each of the above themes. Sample comments from the feedback are included

## Discussion

This study demonstrates that preventive care CDSSs may save clinical time, can be easy to use, and can be useful for clinical practice. A meta-analysis of preventive care CDSSs has already established that CDSSs improve screening, but most CDSSs are not integrated into EMRs and focus on effectiveness instead of workflows and usability [21]. The guiding principle behind this work is the Quadruple Aim for healthcare optimization—improving health outcomes, value, patient experience, and provider experience [22]. This study aims to assess both workflow (task efficiency) and usability (ease of use and usefulness) to assess value and provider experience, respectively.

Value is defined as cost per capita [23] and improved task efficiency can be directly related to reduced cost per capita [6]. Measuring the dollars per capita cost savings is outside the scope of this study. Instead, this study assessed task efficiency showing improvements through preventive care CDSS, which we defined as time savings and fewer actions to complete the given task. This study showed that this preventive care CDSS may provide improved task efficiency, which was accomplished by automatically extracting relevant preventive care data in a single paged summary. This study is unique in that task efficiency relating to CDSS is under-reported in the literature [21]. Extrapolating this time saving across an entire year for an organization could save many hours of work. Physicians could then use this saved time to provide additional healthcare services [2], reclaim lost patient interaction time [10], or tackle copious other EMR tasks [12]. Importantly this improved task efficiency was achieved without impacting decision-making accuracy. Therefore, we suggest that clinical usage of this or similar CDSSs may improve the value of preventive care service delivery by improving task efficiency.

Improving physician EMR efficiency is important since physician burnout is negatively correlated with EMR adoption [10, 12, 24]. Although it is unclear why this association exists, EMR workflows may be the primary culprit. Time motion studies have inconsistently demonstrated the effect of EMR usage on clinician working hours, with reports indicating no impact [14] and others significant extension [11]. It is possible instead that high rates of task switching contribute to perceptions of inefficiency [15]. This CDSS summarizes all data in a single screen that is integrated into the EMR, which we propose may reduce task switching and cognitive loads, leading to high perceived usefulness and ease of use scores. Highly structured EMRs have many data entry fields, buttons, and drop downs, requiring additional user input processes. Each additional action may exacerbate inefficiencies, extend computer interaction times, and reduce direct physician–patient interaction [11]. Accordingly, leveraging this structured data in useful ways via CDSSs may limit the number of user input actions, which do not add value to patient care. In this case those actions are primarily manual chart searches.

In addition to saving time, the use of automated CDSSs may improve the provider experience of care delivery. Participants reported very high usefulness and ease of use scores, which is one measure of provider experience. Participants reported that the CDSS was efficient, comprehensive, organized, and helpful for clinical decision-making. These findings align with the key factors that providers desire in electronic tools including: useful features, easy to use interfaces, efficient designs, valuable information, and practical workflows [25].

Further, this study has also identified several areas for CDSS improvement. Participants did not like the CDSS format, which showed how many months ago a preventive care service was delivered. Instead, participants wanted the specific date of or the number of years since the latest preventive care service, which suggests our approach deviated from participant expectations or usual thought processes. Therefore, we recommend that future CDSS development should understand the intended users’ needs and expectations or more in depth workflow analysis.

As well, participant commented that they would like reminders of the recommended preventive care time intervals. These reminders were deliberately excluded from this CDSS because there is evidence that user interfaces that make decisions for users reduce contemplation, leading to poorer decision-making [26]. Avoiding overly prescriptive decision support has also been recommended as users may inherently trust the information provided to them [27].

This inherent trust in the CDSS could be problematic, which was highlighted in a few participants’ comments about data accuracy. This concern is valid as the accuracy and completeness of EMR data has been shown to be problematic [28]. Reasons for these problems may be that data is missing, incorrectly recorded, out dated [28, 29], has variable terminology, or is misattributed to specific conditions and diagnoses [29]. This CDSS aims to circumvents data quality issues by using data that is automatically important into the EMR by most Ontario laboratories [30] or categorized by the EMR to minimize inaccuracies [18]. This automation may minimize human error and improve data quality. Though challenges can arise when tests have multiple data points in the EMR. For example, Papanicolaou smears data are stored in a variety of ways [18], so the CDSS must account for this issue as was done here.

## Limitations

This study was restricted to one Academic Family Health Team and the CDSS only works with one EMR. Therefore, the generalization of these results to other clinics and EMRs may present challenges. Other clinics may have better or worse workflows altering the time savings and usefulness afforded by this CDSS. As well, this study was conducted in a simulated environment and the benefits highlighted here may not translate to clinical practice.

Since participants were not randomized to do manual chart review or use the CDSS first, participants may have been primed by the first scenario of manual chart review. Accordingly, this may explain some of the time savings in the automated chart review.

## Conclusions

This study demonstrated that CDSSs can leverage EMR data to provide significant time savings without impairing clinical decision-making accuracy. Similar CDSSs may improve EMR workflows and improve the provider experience of care delivery. In part, these time savings and improved experiences may be due to reduced user input requirements and provision of clinically relevant data. Further investigations and CDSS development are required to determine if these findings are applicable to real patient encounters.

## Supplementary Information

**Additional file 1.** Instructions to participants.

**Additional file 2.** Form completed during simulated patient chart analysis.

**Additional file 3.** Perceived Usefulness and Ease of Use Survey completed after the simulated exercise.

**Additional file 4.** Sample Clinical Decision Support System Output.

**Additional file 5.** Clinical Decision Support System Code.

## Data Availability

The datasets used and/or analysed during the current study are available from the corresponding author on reasonable request.
