# Supplementary material for: Improved preventive care clinical decision-making efficiency: leveraging a point-of-care clinical decision support system
Source: BMC Med Inform Decis Mak. 2021 Nov 11;21:315. doi: 10.1186/s12911-021-01675-8 (PMC8588582; doi:10.1186/s12911-021-01675-8)
Supplement: Supplementary file 1 — Additional file 1. Instructions to participants. [file 12911_2021_1675_MOESM1_ESM.docx]

**Appendix A: Instructions to participants.**

**Dynamic EMR Stamps: Perceived Usefulness and Ease of Use in Preventive Care**

Scott Laing PGY2 (Primrose) and Dr Jay Mercer

Thank you for participating in this study. Please see the following information about how to participate. The following exercise is expected to take 15-20 minutes to complete.

**Rationale & Background Information:**

Electronic Medical Records are used to store preventive health data, but without extensive work and maintenance preventive data is not readily available for users to make quick clinical decisions. This study is designed to assess the perceived usefulness and ease of use of a new interface designed to improve access to preventive health data. The primary outcome of this study is to measure users’ perceived usefulness and ease of use of the new interface. Secondary outcomes include efficiency and accuracy of clinical decision-making and qualitative responses to design and function. The data will be used to predict clinical utility and real-world use of the interface in clinical practice.

**Study Objective:**

To assess the perceived usefulness and ease of use of a new, dynamic, preventive care user-interface designed for efficient and accurate clinical decision-making.

**Methodology:**

Participants in the study include physicians, nurses, and other allied health professionals at the Bruyère and Primrose FHT. Each participant will examine two fabricated patient charts as if preparing for a Periodic Health Exam. Participants will identify the preventive health investigations that are due to be completed. Participants will record the previous results of each parameter, identify if repeat testing is due, and record the time required to complete analysis of each individual chart. Please follow the listed instruction below and do not add anything to the fabricated charts.

1. Read through all directions prior to proceeding.
2. Print and review the Preventive Health Data Collection Sheet (page 2) and Perceived Usefulness and Ease of Use Survey.
3. Enter your information at the top of the data collection and survey sheets.
4. Have a stopwatch ready. If you do not have one on your watch/phone, then one can be found here: <https://www.google.ca/search?q=google+stopwatch>
5. Open PS Suites and login.
6. Once logged in click on “Records” to open the chart interface.
7. Press “Ctrl + F” and search for patient last name “FMRSP”
8. Select “FMRSPone, Fake” to open the first chart. A PHE stamp is already inserted in the chart. Please do not add anything to the chart.
9. Start the stopwatch and begin examining the chart. Fill in the following:
   1. when each test was last performed
   2. the results from each test
   3. whether each test is due to be repeated
10. Once completed the first chart stop the stopwatch and record the time in the top right corner.
11. Repeat steps 7 through 10 for patient “FMRSPtwo, Fake” completing the data sheet and recording the time required for completion. The preventive health interface has been inserted in the chart. Please do not add anything to the chart.
12. Once the data collection sheet is completed for each chart, please complete the “Perceived Usefulness and Ease of Use Survey” regarding the new preventive health interface.
13. Please either scan the completed documents and send a PDF to: [slain027@uottawa.com](mailto:slain027@uottawa.com) or staple the sheets together and have the documents delivered to Primrose to be placed in Scott Laing’s mailbox.
