# Supplementary material for: Improved preventive care clinical decision-making efficiency: leveraging a point-of-care clinical decision support system
Source: BMC Med Inform Decis Mak. 2021 Nov 11;21:315. doi: 10.1186/s12911-021-01675-8 (PMC8588582; doi:10.1186/s12911-021-01675-8)
Supplement: Supplementary file 2 — Additional file 2. Form completed during simulated patient chart analysis. [file 12911_2021_1675_MOESM2_ESM.docx]

**Appendix B: Form completed during simulated patient chart analysis**

**Preventive Health Data Collection Sheet**

| **Participant Name:** | | | | |
| --- | --- | --- | --- | --- |
| **Participant Type:** PGY1 □ / PGY2 □ / Staff MD □ / RN □ / Other □ : ______________ | | | | |
| **Performed:** in person □ / remote access □ | | | | |
|  | | | | |
| **Patient:** FMRSPone, Fake | | **Time Required:** _______ (min:sec) | | |
| **Method:** Usual interface (PHE stamp already inserted) | | | | |
| **Metric/Screening** | **Last Done (date/months ago)** | | **Result** | **Due? (Y/N)** |
| BP |  | |  |  |
| BMI |  | |  |  |
| Weight |  | |  |  |
| Waist Circumference |  | |  |  |
| Diabetes |  | |  |  |
| Dyslipidemia |  | |  |  |
| Colorectal Cancer |  | |  |  |
| Cervical cancer |  | |  |  |
| Breast cancer |  | |  |  |
| Osteoporosis |  | |  |  |
| AAA |  | |  |  |
| Immunizations |  | |  |  |
| Others |  | |  |  |
|  | | |  | |
| **Patient:** FMRSPtwo, Fake | | | **Time Required:** _______ (min:sec) | |
| **Method:** New preventive health interface (prevention stamp already inserted in chart) | | | | |
| **Metric/Screening** | **Last Done (date/months ago)** | | **Result** | **Due? (Y/N)** |
| BP |  | |  |  |
| BMI |  | |  |  |
| Weight |  | |  |  |
| Waist Circumference |  | |  |  |
| Diabetes |  | |  |  |
| Dyslipidemia |  | |  |  |
| Colorectal Cancer |  | |  |  |
| Cervical cancer |  | |  |  |
| Breast cancer |  | |  |  |
| Osteoporosis |  | |  |  |
| AAA |  | |  |  |
| Immunizations |  | |  |  |
| Others |  | |  |  |
