# Supplementary material for: Improved preventive care clinical decision-making efficiency: leveraging a point-of-care clinical decision support system
Source: BMC Med Inform Decis Mak. 2021 Nov 11;21:315. doi: 10.1186/s12911-021-01675-8 (PMC8588582; doi:10.1186/s12911-021-01675-8)
Supplement: Supplementary file 3 — Additional file 3. Perceived Usefulness and Ease of Use Survey completed after the simulated exercise. [file 12911_2021_1675_MOESM3_ESM.docx]

**Appendix C: Perceived Usefulness and Ease of Use Survey completed after the simulated exercise**

**Perceived Usefulness and Ease of Use Survey**

| **Participant Name:** | | | | | | | | | | |
| --- | --- | --- | --- | --- | --- | --- | --- | --- | --- | --- |
| **Participant Type:** PGY1 □ / PGY2 □ / Staff MD □ / RN □ / Other □ : ______________ | | | | | | | | | | |
| **Performed:** in person □ / remote access □ | | | | | | | | | | |
| **Perceived Usefulness** |  | **1** | **2** | **3** | **4** | **5** | **6** | **7** |  | **NA** |
| Using this interface in my job would enable me to accomplish tasks more quickly | Unlikely |  |  |  |  |  |  |  | Likely |  |
| Using this interface would improve my job performance | Unlikely |  |  |  |  |  |  |  | Likely |  |
| Using this interface in my job would increase my productivity | Unlikely |  |  |  |  |  |  |  | Likely |  |
| Using this interface would enhance my effectiveness on the job | Unlikely |  |  |  |  |  |  |  | Likely |  |
| Using this interface would make it easier to do my job | Unlikely |  |  |  |  |  |  |  | Likely |  |
| I would find the new system useful in my job | Unlikely |  |  |  |  |  |  |  | Likely |  |
|  |  |  |  |  |  |  |  |  |  |  |
| **Perceived Ease of Use** |  | **1** | **2** | **3** | **4** | **5** | **6** | **7** |  | **NA** |
| Learning to operate this interface would be easy for me | Unlikely |  |  |  |  |  |  |  | Likely |  |
| I would find it easy to get this interface to do what I want it to do | Unlikely |  |  |  |  |  |  |  | Likely |  |
| My interaction with this interface would be clear and understandable | Unlikely |  |  |  |  |  |  |  | Likely |  |
| I would find this interface to be flexible to interact with | Unlikely |  |  |  |  |  |  |  | Likely |  |
| It would be easy for me to become skillful at using this interface | Unlikely |  |  |  |  |  |  |  | Likely |  |
| I would find this interface easy to use | Unlikely |  |  |  |  |  |  |  | Likely |  |
|  |  |  |  |  |  |  |  |  |  |  |
| **List any positive aspects of this interface:** | | | | | | | | | | |
| 1. | | | | | | | | | | |
| 2. | | | | | | | | | | |
| 3. | | | | | | | | | | |
| **List any negative aspects of this interface:** | | | | | | | | | | |
| 1. | | | | | | | | | | |
| 2. | | | | | | | | | | |
| 3. | | | | | | | | | | |
| **How does this interface compare to your usual method of PHE preparation?** | | | | | | | | | | |
|  | | | | | | | | | | |
