# Supplementary material for: Improved preventive care clinical decision-making efficiency: leveraging a point-of-care clinical decision support system
Source: BMC Med Inform Decis Mak. 2021 Nov 11;21:315. doi: 10.1186/s12911-021-01675-8 (PMC8588582; doi:10.1186/s12911-021-01675-8)
Supplement: Supplementary file 4 — Additional file 4. Sample Clinical Decision Support System Output. [file 12911_2021_1675_MOESM4_ESM.docx]

**Appendix D: Sample Clinical Decision Support System Output**

Updated as of Oct 13, 2018

Updated by SL17

Previous Vitals

BP = 118/72 mmHg ; done 29 months ago

BMI = 26.1 kg/m2 ; done 29 months ago

Weight =  71 kg ; done 29 months ago

WC = 82 cm ; done 29 months ago

Immunizations

Td = 87 months

Tdap = 87 months

Pneumococcal = 43 months

Herpes zoster = 43 months

Influenza = 10 months

Previous  Screening

HbA1C = 0.059 ; done 29 months ago

Lipid profile

Total Cholesterol = 4.3 ; done 29 months ago

TG = 1.63 ; done 29 months ago

LDL = 2.5 ; done 29 months ago

HDL = 1.10 ; done 29 months ago

Non-HDL = 3.2 ; done 29 months ago

FOBT done = 42 months ago

FOBT #1 = Negative

FOBT #2 = Negative

FOBT #3 = Negative

Colonoscopy

Result of latest = never done ; done never done months ago

Pap test result (latest) = LSIL ; Report from = 8 months ago ; latest "pap" lab entry = never done

Mammogram BI-RADS = 4 ; Report from = 10 months ago

Bone Mineral Density = 53 months ago

T Score Hip = -0.5

T Score Spine = -0.8

Abdominal Aorta Diameter = never done

Latest abdo US = never done months ago

Latest abdo CT = never done months ago

Vision Reports

Optometry report = never done

Ophthalmology report = never done
