# Supplementary material for: Improved preventive care clinical decision-making efficiency: leveraging a point-of-care clinical decision support system
Source: BMC Med Inform Decis Mak. 2021 Nov 11;21:315. doi: 10.1186/s12911-021-01675-8 (PMC8588582; doi:10.1186/s12911-021-01675-8)
Supplement: Supplementary file 5 — Additional file 5. Clinical Decision Support System Code. [file 12911_2021_1675_MOESM5_ESM.docx]

**Appendix E: Clinical Decision Support System Code**

Updated as of currentDate.default

Updated by currentUserInitials

Last PHE: <65 y pat.Bills.Service_Code"k131a".months_since_latest months ago ; >65 y pat.Bills.Service_Code"k132a".months_since_latest months ago

Previous Vitals

BP = pat.Vitals.Specify_Text_Vital"BP".text_of_latest mmHg ; done pat.Vitals.Specify_Text_Vital"BP".months_since_latest months ago

BMI = pat.Vitals.BMI_[calc_from_Ht_&_Wt].latest_value kg/m2 ; done pat.Vitals.BMI_[calc_from_Ht_&_Wt].months_since_latest months ago

Weight = pat.Vitals.Specify_Text_Vital"Wt".text_of_latest kg ; done pat.Vitals.Specify_Text_Vital"Wt".months_since_latest months ago

WC = pat.Vitals.Specify_Text_Vital"WC".text_of_latest cm ; done pat.Vitals.Specify_Text_Vital"WC".months_since_latest months ago

Immunizations

Td = pat.Immunizations.tetanus_toxoid.months_since_latest months

Tdap = pat.Immunizations.pertussis.months_since_latest months

Pneumococcal = pat.Immunizations.pneumococcal_polysaccharide.months_since_latest months

Herpes zoster = pat.Immunizations.varicella.months_since_latest months

Influenza = pat.Immunizations.influenza_virus_vaccine.months_since_latest months

Previous Screening

HbA1C = pat.Lab_Values.Hemoglobin_A1C_[Hb_A1C].latest_value ; done pat.Lab_Values.Hemoglobin_A1C_[Hb_A1C].months_since_latest months ago

Lipid profile

Total Cholesterol = pat.Lab_Values.Cholesterol_[CHOL].latest_value ; done pat.Lab_Values.Cholesterol_[CHOL].months_since_latest months ago

TG = pat.Lab_Values.Triglycerides_[TG].latest_value ; done pat.Lab_Values.Triglycerides_[TG].months_since_latest months ago

LDL = pat.Lab_Values.Low_Density_Lipoprotein_Cholesterol_[LDL].latest_value ; done pat.Lab_Values.Low_Density_Lipoprotein_Cholesterol_[LDL].months_since_latest months ago

HDL = pat.Lab_Values.High_Density_Lipoprotein_Cholesterol_[HDL].latest_value ; done pat.Lab_Values.High_Density_Lipoprotein_Cholesterol_[HDL].months_since_latest months ago

Non-HDL = pat.Lab_Values.Non-HDL_cholesterol_[Non-HDL].latest_value ; done pat.Lab_Values.Non-HDL_cholesterol_[Non-HDL].months_since_latest months ago

FOBT done = pat.Lab_Values.Stool_Occult_Blood_[Stool_Occult_Blood].months_since_latest months ago

FOBT #1 = pat.Lab_Values.Stool_Occult_Blood_[Stool_Occult_Blood].latest_value

FOBT #2 = pat.Lab_Values.Stool_Occult_Blood_#2_[Stool_Occult_Blood_#2].latest_value

FOBT #3 = pat.Lab_Values.Stool_Occult_Blood_#3_[Stool_Occult_Blood_#3].latest_value

Colonoscopy

Result of latest = pat.Vitals.Specify_Text_Vital"ColonoscopyResult".text_of_latest ; done pat.Diagnostic_Test_Reports.Colonoscopy.months_since_latest months ago

Repeat after = pat.Vitals.Specify_Text_Vital"ColonoscopyDue".text_of_latest years

Pap test result (latest) = pat.Vitals.Specify_Text_Vital"PapResult".text_of_latest ; Report from = pat.Diagnostic_Test_Reports.Pap_Test_Report.months_since_latest months ago ; latest "pap" lab entry = pat.Lab_Values.Pap_Smear_[Pap_Smear].latest_value

Mammogram BI-RADS = pat.Vitals.Specify_Text_Vital"BI-RADS".text_of_latest ; Report from = pat.Diagnostic_Imaging.Mammogram.months_since_latest months ago

Bone Mineral Density = pat.Diagnostic_Imaging.Bone_Densitometry.months_since_latest months ago

T Score Hip = pat.Vitals.Specify_Numeric_Vital"T-scoreHip".latest_value ; pat.Vitals.Specify_Numeric_Vital"T-scoreHip".months_since_latest months ago

T Score Spine = pat.Vitals.Specify_Numeric_Vital"T-scoreSpine".latest_value ; pat.Vitals.Specify_Numeric_Vital"T-scoreSpine".months_since_latest months ago

Vision Reports

Optometry report = pat.Consultant_Letter.Optometry.months_since_latest months ago

Ophthalmology report = pat.Consultant_Letter.Ophthalmology.months_since_latest months ago
